# A network analysis of subjective well-being in Chinese high school students

**DOI:** 10.1186/s12889-023-16156-y

**Published:** 2023-06-27

**Authors:** Shiwei Wang, Siqi Zhao, Yan Guo, Chengjing Huang, Pei Zhang, Lu She, Bing Xiang, Jing Zeng, Feng Zhou, Xinyan Xie, Mei Yang

**Affiliations:** 1 Research Center for Health Promotion in Women, Youth and Children, School of public health, Wuhan University of science and technology, Wuhan, 430065 Hubei Province China; 2 Wuhan centers for disease control and prevention, Wuhan, 430022 Hubei Province China

**Keywords:** Network analysis, High school students, Subjective well-being

## Abstract

**Background:**

The psychological situation of high school students during adolescence is not promising, and the most obvious manifestation is the lack of subjective well-being (SWB). This network analysis presents a model of the interaction and correlation between different items of SWB, identifying the most central items for high school students.

**Methods:**

Through offline and online surveys, 4,378 questionnaires were sent out and finally 4,282 Chinese high school students were available. The response rate was 97.807%. The study used the eLASSO method to estimate the network structure and centrality measures. This algorithm used the EBIC to select the best neighbor factor for each node.

**Results:**

The average age for high school students was 16.320 years old and the average SWB score was 76.680. The distribution of SWB between male and female students was significant different (*P* < 0.001). S8 (Have you been anxious, worried, or upset) was the node with the highest strength and expected influence. The network structure and centrality remained stable after discarding 75% of the sample at random. Except for S15 (How concerned or worried about your health have you been), all nodes were positively correlated with each other (*P* < 0.01). The network structure of SWB was similar for female and male students (network strength: 8.482 for male participants; 8.323 for female participants; *P* = 0.159), as well as for rural and urban students (network strength: 8.500 for rural students; 8.315 for urban students; *P* = 0.140).

**Conclusion:**

Targeting S8 (Have you been anxious, worried, or upset) as a potential intervention target may increase high school students’ SWB effectively.

**Supplementary Information:**

The online version contains supplementary material available at 10.1186/s12889-023-16156-y.

## Introduction

High school students, who are in the puberty stage of rapid psychological and physical development, have been proved that most of them are not in an optimistic psychological condition. And the lack of subjective well-being (SWB) is one of the more obvious manifestations [1–3]. SWB is defined as people’s relevant evaluation of their holistic quality of life and emotions in several dimensions in accordance with their own judgment criteria as a foundation, which leads to satisfaction with life and the various emotions that result from it [1, 4, 5]. Current researches are increasingly emphasizing the positive effects of SWB on physical and mental health, longevity, prevention of mental illness, and attitude toward life [6, 7]. And the existed evidences have shown that high school students with higher SWB have higher levels of mental health, higher levels of creativity, and better relationships with others, while high school students with low SWB have lower levels of mental health and quality of life [5, 8, 9]. Therefore, it is particularly important to study the SWB of high school students, especially to identify the most prominent issues in SWB that affect high school students, in order to provide more targeted interventions and coping strategies to improve their SWB and then promote their mental health.

Network analysis is a new method for investigating the complex, dynamic relationships between individual psychiatric symptoms, which is oriented towards the combination and interaction of symptoms [10, 11]. And the network analysis can perform the strength and nature of the associations among psychiatric symptoms [12, 13].In the network structure, “nodes” represent symptoms and “edges” represent inferred statistical associations between symptoms. The stability of the network structure is tested by contrasting the differences between nodes and edges. Network structure of psychotic-like experiences in adolescents: links with risk and protective factors [14, 15]. The “centrality index” is usually assessed by three metrics: strength, closeness and betweenness, and is often used to infer the importance of each node in the network [14, 16, 17]. The strength of a node is the sum of the absolute values of its connections to other nodes in the network. The closeness of a node is the average shortest path between a given node and the rest of the nodes in the network. Nodes with higher closeness are more closely connected to the rest of the network [17]. And betweenness is the number of times a given node lies on the shortest path between two other nodes [18, 19]. In the analysis of psychological interventions, the network analysis operates on the underlying mechanisms of intervention efficacy by revealing complex patterns of nodal associative change [15, 20, 21]. The application of the network analysis in the study of high school students’ SWB may facilitate us to be able to identify the most important internal factors.

According to our knowledge, a variety studies have been conducted to evaluate the effects of external factors, such as self-esteem [1, 22, 23], self-control [24–26], depression, anxiety [6, 27] and personality traits, on SWB of high school students. But few articles explore the interactions of internal factors of SWB. Although, Ventura-León et al. [28] have done a network analysis of SWB related to depression in college students, the most salient issues affecting SWB have not been investigated. Accordingly, it is necessity to have a more in-depth understanding of the SWB profile in the population of high school students.

Consequently, this study aimed to establish the network structure of SWB of Chinese high school students. We specifically attempted to: (1) identify the interrelationships between the nodes of each entry; (2) identify the core nodes of the SWB network structure; and (3) examine the stability of the network structure.

## Methods

**Participants**

In this study, the cluster sampling method was used. According to the sample size calculation formula $n \approx \frac{{{{\left( {{z_{a/2}}} \right)}^2}{\sigma ^2}}}{{{E^2}}}$, where *z*_*a*/2_ is the corresponding value of confidence level, ${\sigma }^{2}$ is the variance, and E is the tolerance error, the sample size was calculated by taking a=0.05, *z*_*a*/2_=1.96, and E=1.5%, and the sample size was calculated to be 4268.

During March to May 2021, using the methods of cross-sectional investigation, 4378 students from 2 high schools in Wuhan, Hubei Province, China were clustered selected as the research participants according to the following criteria: internal students, no cognitive impairment, signed informed consents and volunteer to participate in this study. The questionnaires were retrieved by the trained workers. Considering the different penetration of mobile phones and convenience, both the paper and electronic questionnaires were used. After clarifying the significance, confidentiality and precautions of this survey, all participants filled out the questionnaire anonymously. After the questionnaires were collected, relevant reviews were conducted immediately to exclude invalid questionnaires such as no answers and blanks.

## Variables and measures

### Subjective well-being

In this study, the levels of SWB were measured using the General Well-Being Schedule, which was developed by Fazio [29]. The scale has been widely used and has good reliability and validity [3]. General Well-Being Schedule consists of 18 items, of which items 1–14 are scored on a 6-point scale and 15–18 on a 10-point scale. Items 1, 3, 6, 7, 9, 11, 13, 15 and 16 are reverse scores, while the rest are scored positively. And this scale can divide into six dimensions: satisfaction and interest in life, health concerns, energy, a melancholy or cheerful mood, emotional and behavioral control, relaxation and tension. The sum of the option scores for each item represents the total score, and the higher the score, the stronger the SWB. The test-retest reliability coefficient of the scale in this study is 0.850, and the correlation coefficient between the subscale and the total scale is 0.560 ~ 0.880. The Cronbach’s α in this study was 0.780, and factor analysis showed a KMO of 0.912.

### Network structure and centrality measures analysis

All analyses were conducted using R (Version 4.2.1) [30]. Mean, standard deviation (SD), kurtosis, skewness of all the SWB items were inspected. The “Goldbrick” function from the “networktools “package [31] was used to check the data for potentially redundant nodes. The R packages “bootnet” (version 1.4.3) [32] and “qgraph” (version 1.6.5) [33] were used to estimate and visualize the network.

The network structure of SWB was estimated using the enhanced Least Absolute Shrinkage and Selection Operator (eLASSO) method. The eLASSO method can be used to shrink all edges so that small edges become zero-weighted edges to obtain a more stable and interpretable network to judge and measure well the sensitivity and specificity of finding true edges [34]. This algorithm used penalty parameters to obtain sparsity, and used the Extended Bayesian Information Criterion (EBIC) (a measure of goodness of fit) to select the best neighbor factor set for each node (one node represents a single symptom) [11, 35]. EBIC minimization can better discover the network model of real connections. The EBIC-based lasso algorithm is one of the most specific algorithms that can distinguish false connections from real connections and does not estimate those false connections (i.e., connections that do not exist in the real network) [36, 37]. It effectively avoids multicollinearity between entries by filtering the variables and simplifies the network structure model [38].

When each node was connected to another node through edges with different weights (an edge represents a link between two nodes), the network automatically constructed a strength index of the direct association. Fruchterman-Reinfold algorithm was applied to visualize the network. In the network, the thickness of an edge represented the strength of the association between nodes and the color of the edge indicated the direction of the association (blue indicating positive association and red indicating negative association). The thicker the edge, the greater the correlation between the two nodes. Nodes with stronger and more frequent associations with another node were positioned closer together and were more concentrated in the network. Network analysis provided quantitative centrality symptoms for each node based on the distinct configuration of the network [35].

According to previous studies, strength centrality was more stable than closeness and betweenness [10, 39]. Strength centrality represented the sum of the edge weights of each node (e.g., correlation coefficient), reflecting the likelihood that activation of a symptom might lead to activation of other symptoms [40]. The “qgraph” package was used in this study to take strength and closeness as centrality indexes. The R package “mgm“ [34] was also used to estimate the expected influence of each node. The higher the expected influence value, the higher the centrality of the network structure. In this study, centrality measures were reported as standardized values (z-scores) [17, 19].

We used the “Network Comparison Test” (version 2.2.1) [41] to check whether there were differences in the network structure of SWB in subgroups based on gender and place of residence through the Holm multiple comparison correction network comparison test (NCT).NCT based on three invariance measures: network structure invariance, global strength invariance (the sum of all edges in the network) and edge strength invariance [42].

### Estimation of network accuracy and stability

To assess the robustness of the network structure, the R-package “bootnet” was used to evaluate the stability and accuracy of the network. The stability of the network was estimated using the case dropping bootstrap procedure [12, 13]. The network was considered stable if the majority of samples could be excluded from the dataset without observing significant changes in the centrality index of the nodes. Stability was represented graphically and quantified by calculating the correlation stability coefficient (CS-C) [11, 15, 41]. The non-parametric bootstrap was used to calculate its confidence interval (CI) to estimate the accuracy of the edge weight [43]. Observations were randomly resampled to generate multiple new datasets from which 95% CI were calculated. In this network analysis, we performed 1000 permutations and used bootstrap difference tests to assess differences in network attributes [16, 17, 39].

## Results

### Study sample

A total of 4378 high school students were enrolled to participate in this study. Among them, 96 questionnaires with missing information, which affected the data analysis, were finally excluded. Finally, 4282 students were included with an effective recovery rate of 97.807%.

As presented in Table 1, the average age of the subjects was 16.320 (SD = 0.536) years old, and the gender distribution was basically equal (46.287% were male). The average total SWB score of the whole sample was 76.680 (SD = 13.895). The distribution of SWB in male and female students were significant different (*P* < 0.001), while the SWB differences in differnet grades and residences were not significant (*P* > 0.05).The means, SD, skewness and kurtosis of SWB items were shown in Supplementary Table 1.

**Table 1:** Characteristics of the participants

Variables	N = 4282(n, %)	SWB($\bar x$, SD)	*P*
**All**	**—**	76.680 (13.895)	
**Age**	**—**	16.320 (0.536)	
**Gender**			**< 0.001**
Male	1982 (46.287%)	78.020(14.001)	
Female	2300 (53.713%)	75.540(13.640)	
**Residence**			0.188
Rural	1012 (23.634%)	76.180(13.570)	
Urban	3270 (76.366%)	76.840(13.941)	
**Grade**			0.332
Tenth grade	1936 (45.213%)	76.960(14.050)	
Eleventh grade	1637 (38.230%)	76.630(13.821)	
Twelfth grade	709 (16.557%)	76.060(13.430)	

### Network structure and centrality measures analysis

The information and redundancy of scale items were tested, and no item was rated below the average information level, indicating that no item was redundant when related to other items (< 25% statistically different correlation). Therefore, all items of the SWB were included in the network analysis.

**Fig. 1:**
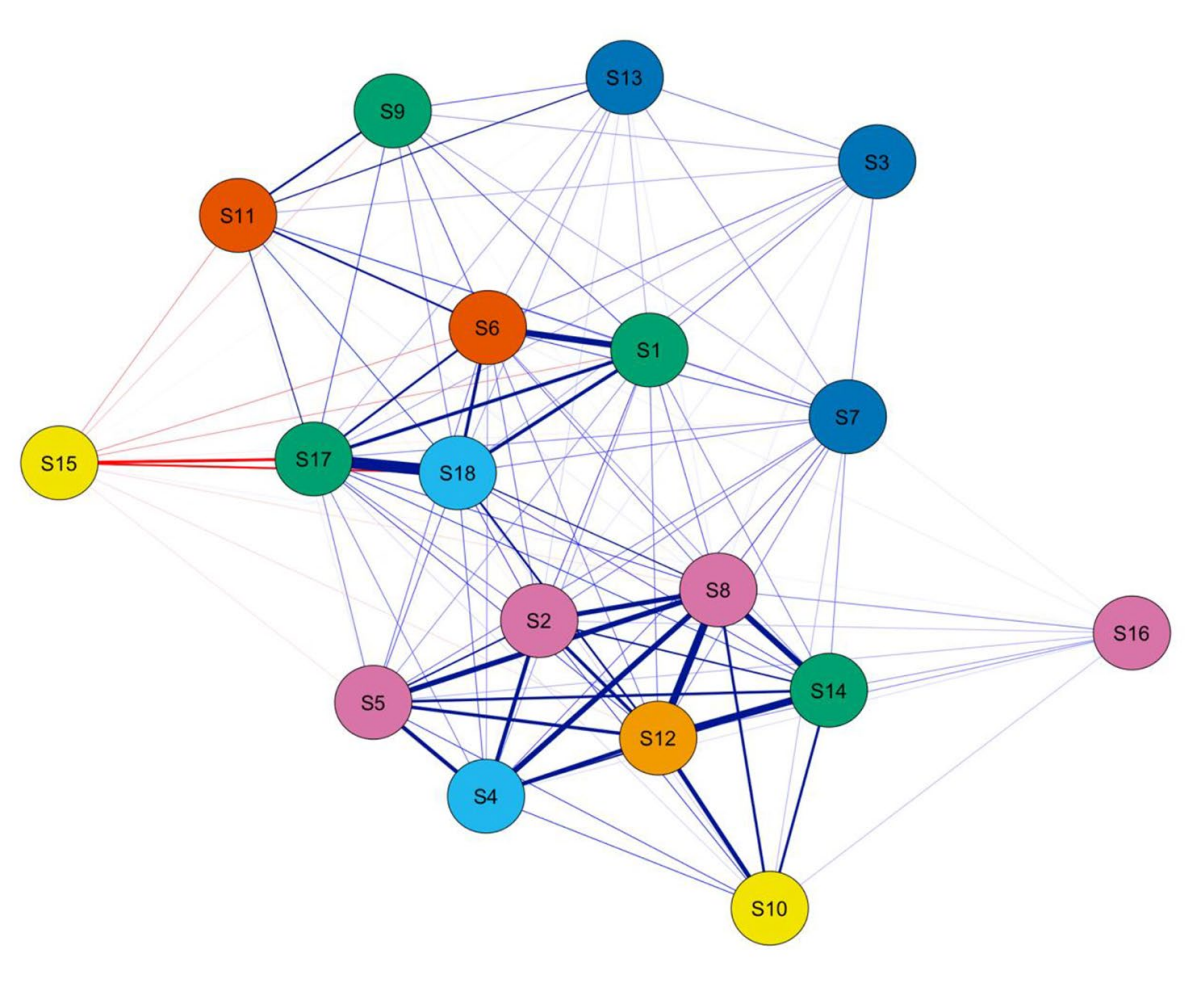
Network analysis of SWB of high school students **Note**: S1: How have you been feeling in general, S2: Have you been bothered by nervousness or you “nerves”, S3: Have you been in firm control of your behavior, thoughts, emotions or feeling, S4: Have you felt so sad, discouraged, hopeless, or had so many problems that you wondered if anything was worthwhile, S5: Have you been under or felt you were under any strain, stress, or pressure, S6: How happy, satisfied, or pleased have you been with your personal life, S7: Have you had any reason to wonder if you were losing your mind, or losing control over the way you act, talk, think, feel, or of your memory, S8: Have you been anxious, worried, or upset, S9: Have you been waking up fresh rested, S10: Have you been waking up fresh and rested, S11: Has your daily life been full of things that were interesting to you, S12: Have you felt down-hearted and blue, S13: Have you been feeling emotionally stable and sure of yourself, S14: Have you felt tired, worn out, used-up, or exhausted, S15: How concerned or worried about your health have you been How much do you care or worry about your health, S16: How relaxed or tense have you been, S17: How much energy, pep, vitality have you felt, S18: How depressed or cheerful have you been.

**Fig. 2:**
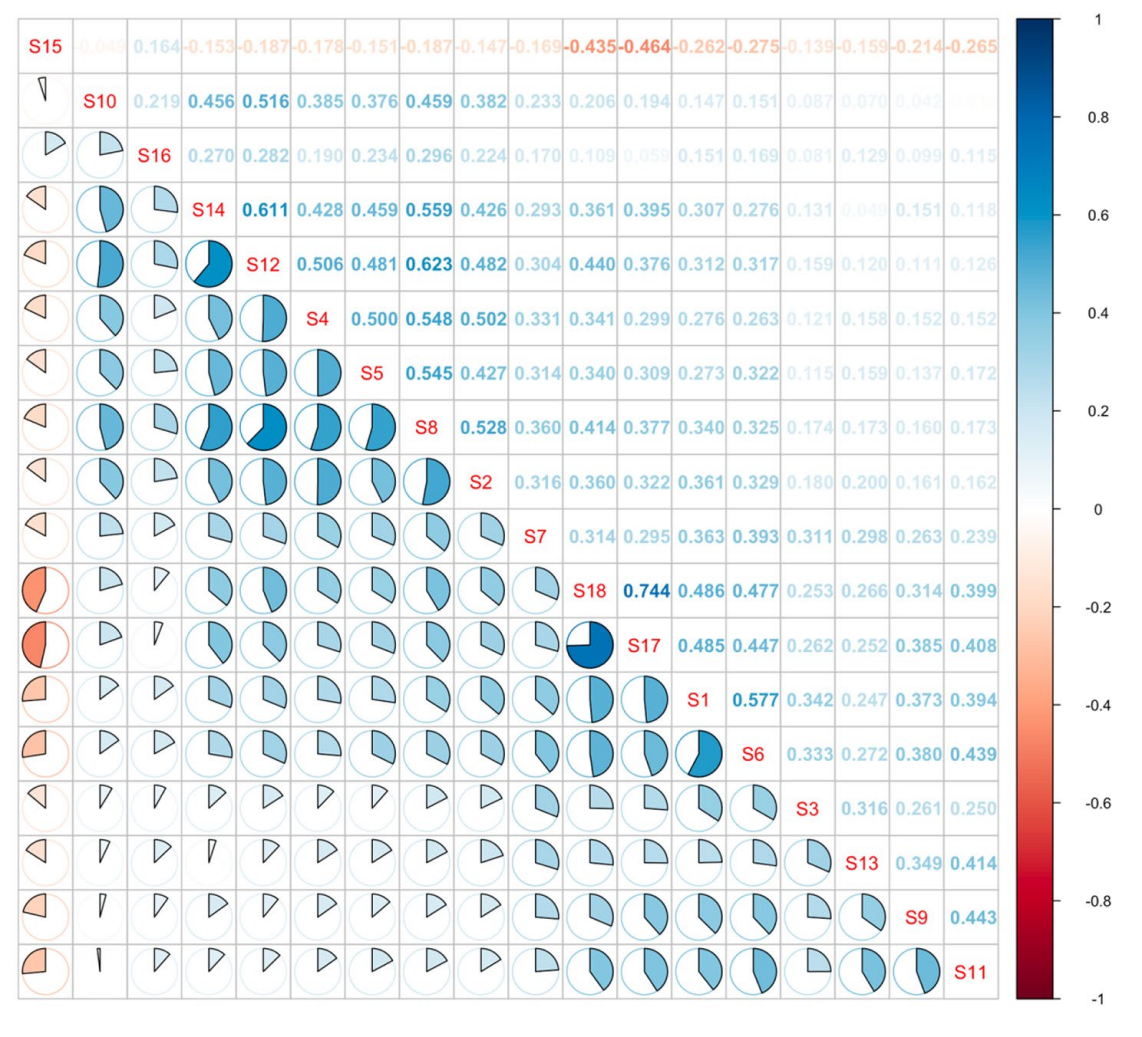
Correlation matrix of the SWB items Note: *P*<0.01

Network analysis of SWB was shown in Fig. 1. S15 (How concerned or worried about your health have you been) showed negative correlation with other nodes except S16 (How relaxed or tense have you been) (*r* = 0.164, *P* < 0.01). And between all the remaining nodes, the correlations were positive. S1 (How have you been feeling in general), S6 (How happy, satisfied, or pleased have you been with your personal life), S17 (How much energy, pep, vitality have you felt) and S18 (How depressed or cheerful have you been) had the strongest correlations, and the correlation coefficient between S17 and S18 was 0.744 (*P* < 0.01). There were strong associations between S2 (Have you been bothered by nervousness or you “nerves”), S4 (Have you felt so sad, discouraged, hopeless, or had so many problems that you wondered if anything was worthwhile), S5 (Have you been under or felt you were under any strain, stress, or pressure), S8 (Have you been anxious, worried, or upset), S12 (Have you felt down-hearted and blue) and S14 (Have you felt tired, worn out, used-up, or exhausted), and S8 and S12 with the correlation coefficient of 0.623 (*P* < 0.01). Pearson correlations between variables were reported in Fig. 2.

**Fig. 3:**
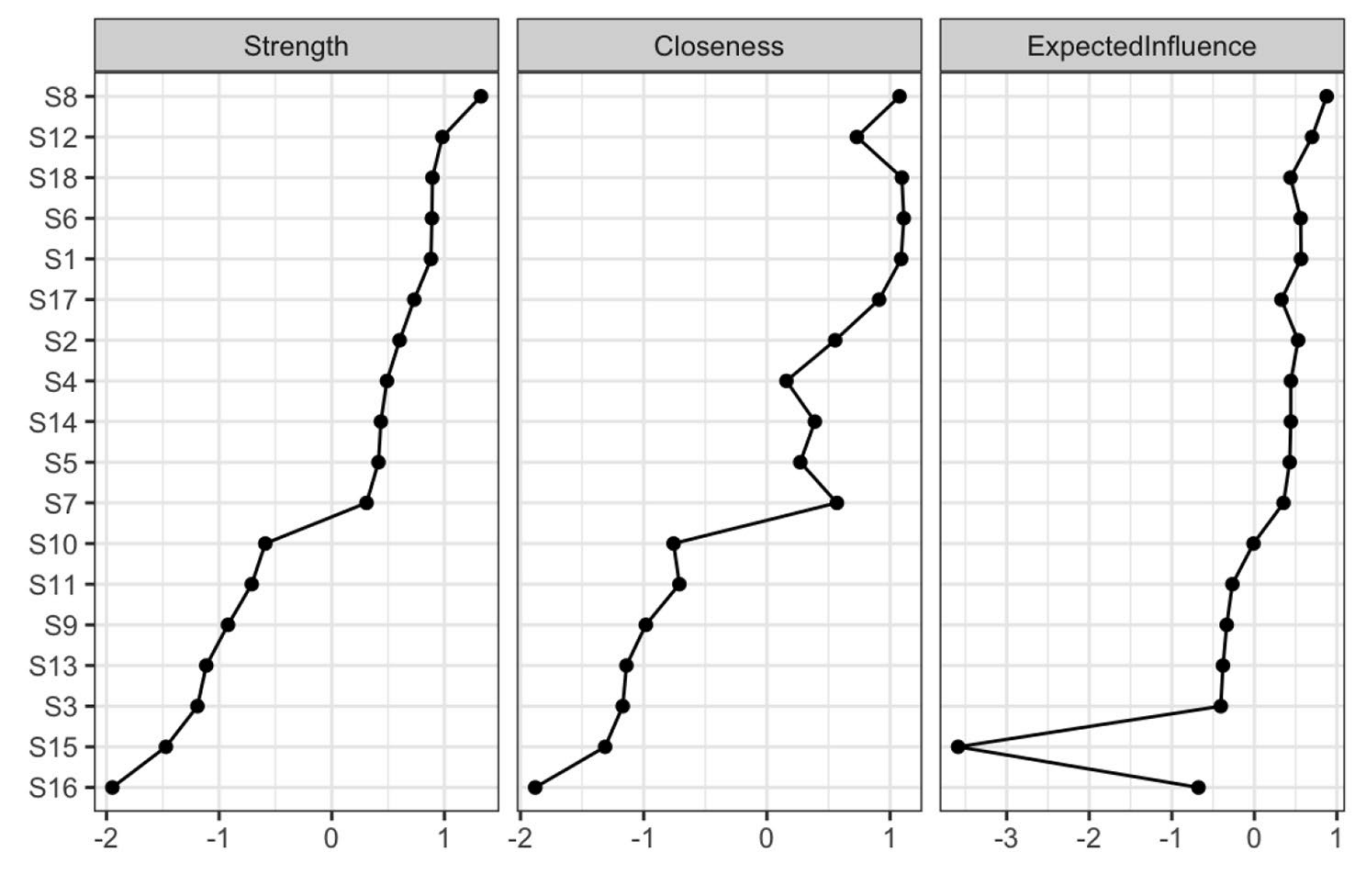
Strength, closeness and expected influence of network structure of SWB

The network centrality indexes were shown in Fig. 3. The stability coefficient of the strength centrality of the overall network model reached 0.75, which was greater than 0.5, indicating that the stability of the model was satisfactory. Regarding strength centrality in the network of SWB, S8 (Have you been anxious, worried, or upset) was the strongest item connected with other nodes on the shortest path, followed by S12 (Have you felt down-hearted and blue) and S18 (How depressed or cheerful have you been). Closeness centrality data suggested that S6 (How happy, satisfied, or pleased have you been with your personal life) was the most central performance, followed by S1 (How have you been feeling in general) and S18(How depressed or cheerful have you been). At the same time, S8 (Have you been anxious, worried, or upset) was the core node in expected influence. This showed that S8 was the most central node in the SWB network for high school students, and played a key role in the whole network.

### Estimation of network accuracy and stability

**Fig. 4:**
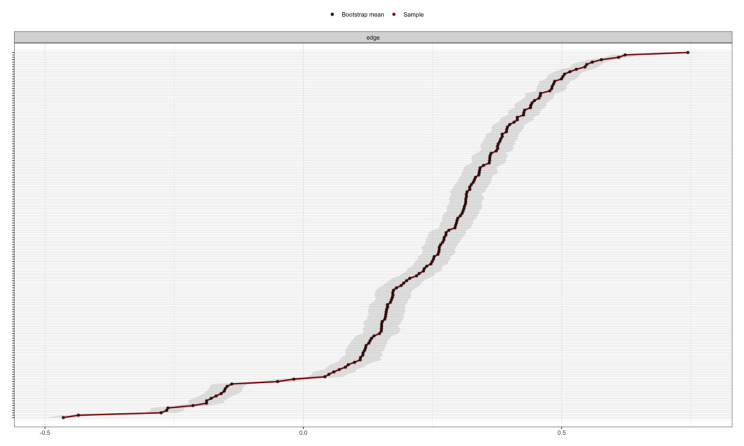
The accuracy of edge weights in SWB network **Note**: the red line represents the edge weight value of the sample in this study, and the black line represents the average edge weight value evaluated by the self-service method. The grey area represents the confidence interval calculated by the self-service method. The abscissa represents the regularized partial correlation coefficient, and the ordinate represents the edge in the network.

As shown in Fig. 4, in the edge weight accuracy chart, the 95% CI of the edge weight value was narrow, indicating that the evaluation of the edge weight value of the SWB network structure diagram was accurate.

**Fig. 5:**
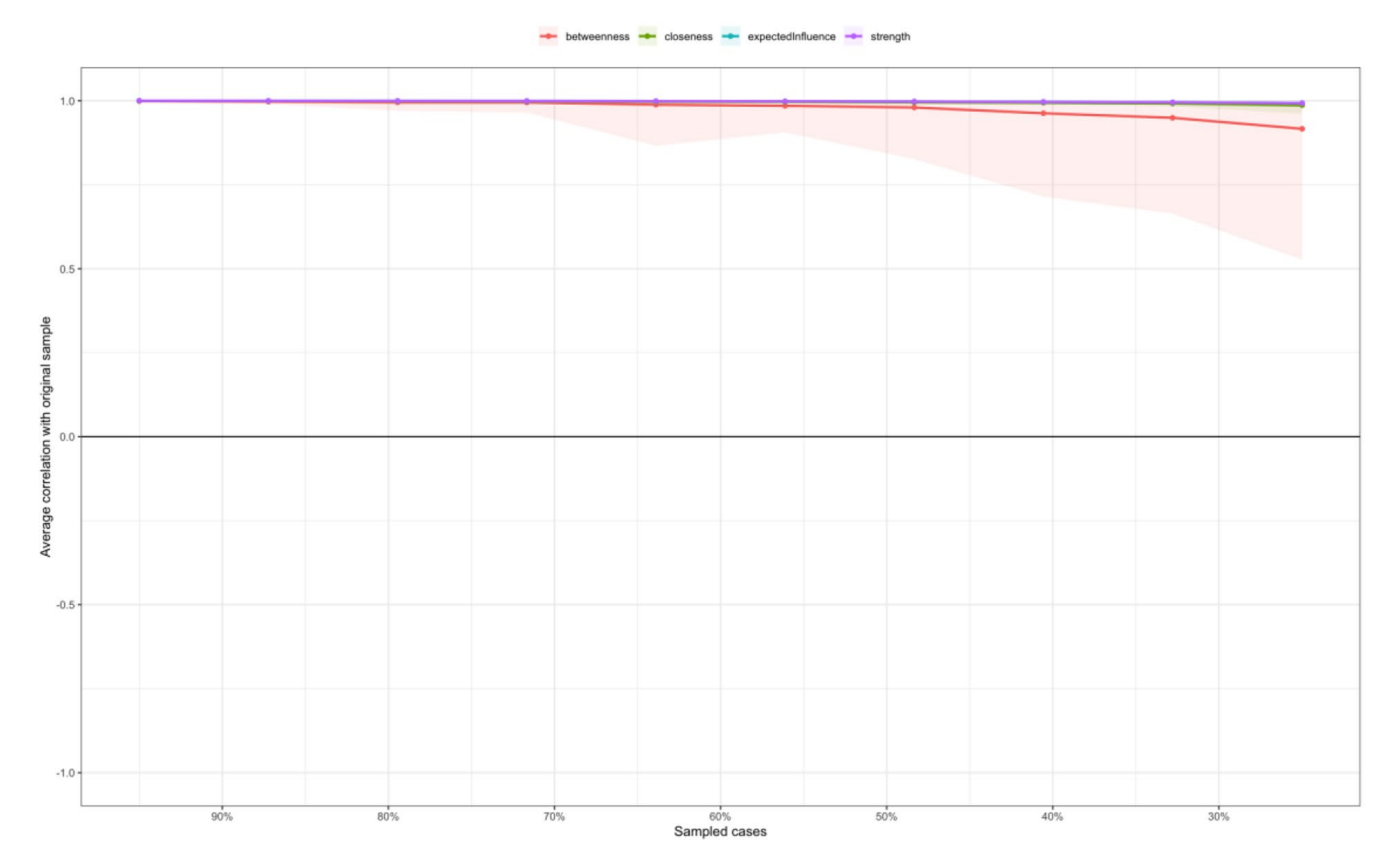
Stability of centrality indices by case dropping subset bootstrap **Note**: The x-axis represents the percentage of cases of original sample used at each step. The y-axis represents the average of correlations between the centrality indices from the original network and the centrality indices from the networks that were re-estimated after dropping increasing percentages of cases. Each line indicates the correlations of betweenness, strength, closeness, expected influence, while areas indicate 95% CI.

For stability of the network analysis, strength, closeness and expected influence had an excellent level of stability (i.e., CS-C = 0.75) which indicated that 75% of the sample could be dropped and then the structure of the network did not significantly change (Fig. 5).

The bootstrap difference test showed that most of the comparisons between strength and closeness of each node were statistically significant (supplementary Fig. 1 and 2). The results of the edge weight differences were shown in supplementary Fig. 3, and the comparisons between most edge weights were statistically significant.

### Network comparison test

Comparison of the network structure model between female (n = 2300) and male (n = 1982) high school students did not reveal significant differences in overall network strength (network strength: 8.482 for male participants; 8.323 for female participants; *P* = 0.159) and edge weights (M = 0.156, *P* = 0.520, supplementary Fig. 4-6). The maximum value of all differences between male and female students was not significant (*P* = 0.560).

We also compared the network structure of SWB by rural (n = 1012) and urban (n = 3270), and the result was not significant different (network strength: 8.500 for rural students; 8.315 for urban students; *P* = 0.140) and edge weights (M = 0.158, *P* = 0.630, supplementary Fig. 7-9). Similar to the strength of the overall network structure, S8 (Have you been anxious, worried, or upset) was the strongest item.

## Discussion

As far as we know, this is the first study to describe the network structure of SWB in Chinese high school students. We found that there were differences in the strength, closeness and expected influence among each item. Among them, S8 (Have you been anxious, worried, or upset) had the highest centrality which was the core node of the network structure, and is more likely to affect other nodes. This means that the activation of the S8 item may activate other projects associated with it, which in turn activates the entire SWB network broadly. That is, taking S8 (Have you been anxious, worried, or upset) as the intervention target may maximize the overall mental health level of high school students.

In addition, we also discovered a number of other more centralized items were S12 (Have you felt down-hearted and blue) and S18 (How depressed or cheerful have you been). These items were all negative emotions, which were most likely to lead to low subjective happiness level of high school students. This was similar to the findings of Paciello et al. [44], who found an association between self-efficacy and SWB, while self-efficacy was associated with regulating negative emotions, getting help from others, and self-regulating learning [45]. Negative emotions was defined as a basic subjective experience of being depressed and in an unpleasant situation, including emotional states such as depression, anxiety, anger, sadness, etc [46, 47].

In fact, negative emotions is an important part of well-being, and how to guide high school students to properly self-regulate their emotions is a very important part. The daily experience of individuals with positive emotions will increase with the passage of time, thus making individuals better adapt to social changes. The Stress-vulnerability hypothesis holds that when an individual is exposed to a high-pressure environment, positive factors will lose their protective effect [48]. The " immune” effect of positive factors on SWB of high school students may be lost when unmanageable stress, anxiety and depression are felt. Anxiety, worry and frustration are inevitable in the growth process of high school students, and it is important for families and schools to focus on the psychological health of high school students and guide them on how to cope with such negative emotions, which in turn will enhance the SWB of individuals.

The network structure of male and female students was similar. S8 (Have you been anxious, worried, or upset) also had the highest centrality among the network structure for boys and girls. Except S9 (Have you been waking up fresh rested), S10 (Have you been waking up fresh and rested) and S15 (How concerned or worried about your health have you been) nodes had the same strength for male and female students, the strength of other nodes was higher in girls than in boys. This may be related to gender role orientation and physical and mental development differences between male and female students. When experiencing stressful events such as setbacks, boys have high self-esteem and are reluctant to take recourse to deal with them compared to girls, which in turn makes the node strength of girls’ SWB network structure higher. Whether boys or girls, they should be encouraged to learn how to cope with negative emotions such as anxiety, worry and depression, reduce their negative effects and promote their psychological well-being.

Given the critical role of high centrality of network structure in previous studies, our study’s findings contribute to the development of an intervention to enhance the SWB of high school students [20]. That is, families, schools, and society should develop active and effective preventive measures to relieve common tension, stress, and anxiety events of high school students, in order to improve their well-being and mental health. Firstly, school authorities should incorporate SWB improving into the psychology curriculum, and equip students with the skills to cope with negative emotions. Secondly, parents should pay attention to their offspiring’s emotions, enhance communication with them, try to understand and accompany them, and provide necessary psychological supports. Thirdly, the society should strengthen mental health promotion and assistance programs. Most importantly, students should strengthen their self-relieve ability through appropriate physical exercise, healthy eating habits, adequate sleep, good interpersional relationship skills and so on. Finally, the influence of negative emotions can be effectively de-escalated, which in turn can enhance subjective well-being.

Although this study has important theoretical and practical significance, it also has some limitations and points out the direction for further exploration in the future. First of all, this study mainly collected data from Chinese teenagers. Due to the fact that part of third-grade students was unable to participate in the reform project due to going out for practice, the proportion of third-grade students was relatively small. The applicability of this model in senior grades still needs further verification. Secondly, although the sample size was large, the use of self-reporting to collect research data might be subject to information bias. And the generalization of the results of this study may be limited.

## Conclusion

Most previous studies have explored the relationships between SWB and other external variables, but little was known about the associations between internal variables of SWB. Therefore, this study aimed to use network analysis to explore the relationship between internal variables of SWB by identifying the most core items of SWB among high school students. The results showed that S8 (Have you been anxious, worried, or upset) was the most central item in the network. Through this deeper analysis, using S8 (Have you been anxious, worried, or upset) as a potential intervention target may lead to greater improvement in high school students’ SWB and thus mental health.

## Electronic supplementary material

Below is the link to the electronic supplementary material.

Supplementary Material 1

## Data Availability

All data generated or analysed during this study are included in this published article.

## List of Abbreviations

CI: Confidence interval
CS-C: Correlation stability coefficient
EBIC: The Extended Bayesian Information Criterion
eLASSO: The enhanced Least Absolute Shrinkage and Selection Operator
SWB: Subjective well-being
