# Supplementary material for: A network analysis of subjective well-being in Chinese high school students
Source: BMC Public Health. 2023 Jun 27;23:1249. doi: 10.1186/s12889-023-16156-y (PMC10304267; doi:10.1186/s12889-023-16156-y)
Supplement: Supplementary file 1 — Supplementary Material 1 [file 12889_2023_16156_MOESM1_ESM.docx]

**Supplementary Materials**

**Supplementary Table 1：The means, SD, skewness and kurtosis of SWB items.**

**Supplementary Figure 1：Estimation of node strength differences by bootstrapped difference test.**

**Supplementary Figure 2：Estimation of node closeness differences by bootstrapped difference test.**

**Supplementary Figure3: Estimation of edge weight differences by bootstrapped difference test.**

**Supplementary Figure 4：Network Analysis of Subjective Well-being of high School Boys.**

**Supplementary Figure 5：Network Analysis of Subjective Well-being of high School girls.**

**Supplementary Figure 6：Node strength based on subgroups by gender.**

**Supplementary Figure 7：Network analysis of rural high school students' subjective well-being.**

**Supplementary Figure 8：Network analysis of urban high school students' subjective well-being.**

**Supplementary Figure 9：Node strength based on subgroups by residence.**

**Supplementary Table S1: The means, SD, skewness, and kurtosis (n = 4,282)**

| **Item** | **Means** | **SD** | **Skewness** | **kurtosis** |
| --- | --- | --- | --- | --- |
| SWB-1 | 4.080 | 1.254 | 0.178 | -0.800 |
| SWB-2 | 3.640 | 1.003 | -0.395 | -0.116 |
| SWB-3 | 4.410 | 1.050 | -0.565 | 0.486 |
| SWB-4 | 4.230 | 1.295 | -0.700 | 0.146 |
| SWB-5 | 3.460 | 1.102 | -0.444 | -0.264 |
| SWB-6 | 3.360 | 1.090 | 0.159 | -0.853 |
| SWB-7 | 3.920 | 1.004 | -0.692 | -0.182 |
| SWB-8 | 4.310 | 1.231 | -0.735 | 0.562 |
| SWB-9 | 3.510 | 1.356 | 0.247 | -0.521 |
| SWB-10 | 4.970 | 1.219 | -1.296 | 1.331 |
| SWB-11 | 3.510 | 1.372 | 0.075 | -0.865 |
| SWB-12 | 4.430 | 1.260 | -0.753 | 0.117 |
| SWB-13 | 3.950 | 1.416 | -0.309 | -0.869 |
| SWB-14 | 4.170 | 1.219 | -0.521 | -0.287 |
| SWB-15 | 2.630 | 2.845 | 0.999 | 0.222 |
| SWB-16 | 4.870 | 2.688 | 0.121 | -0.443 |
| SWB-17 | 6.520 | 2.677 | -0.476 | -0.327 |
| SWB-18 | 6.720 | 2.572 | -0.491 | -0.2 |


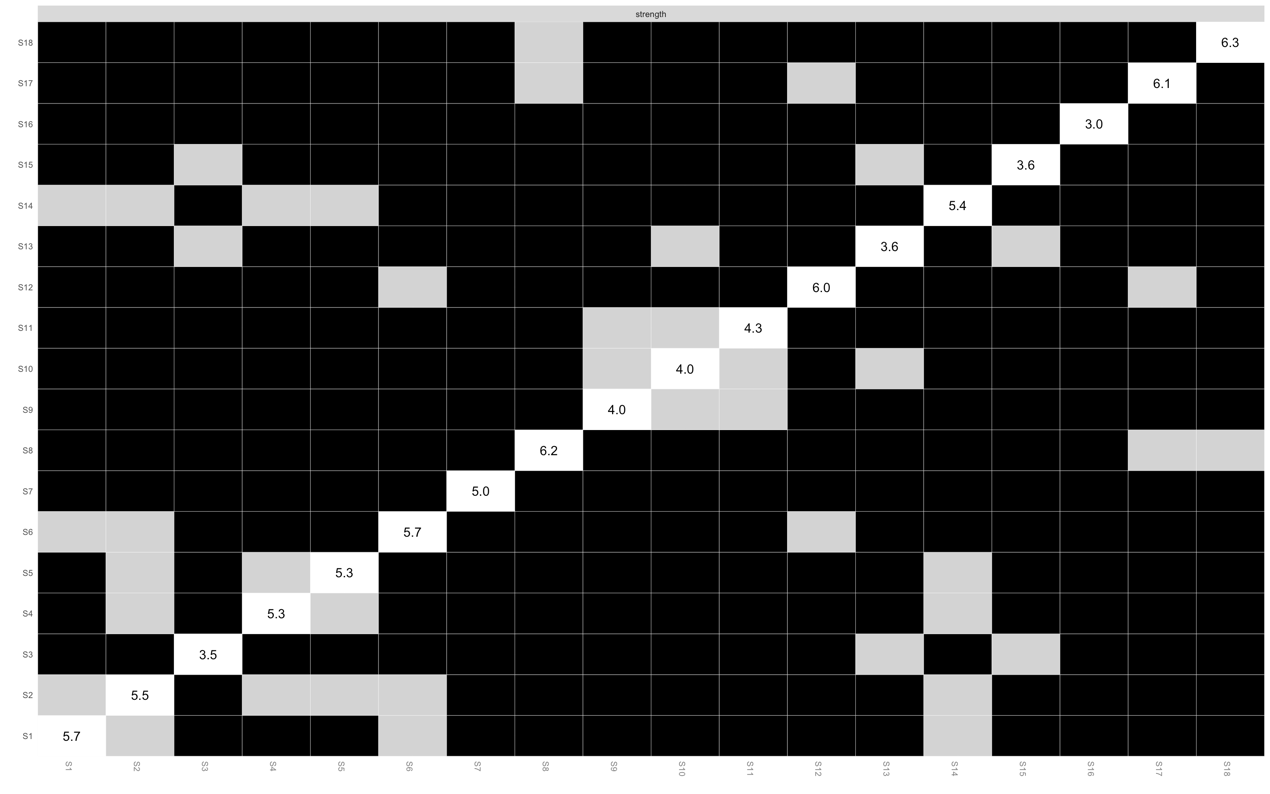


**Supplementary Figure 1: Estimation of node strength differences by bootstrapped difference test**

**Note:** Nonparametric bootstrapped difference test for strength. Gray boxes indicate no difference between nodes, whereas black boxes indicate significant difference (α = 0.05). Values reported in the diagonal represent the strength values of each node.


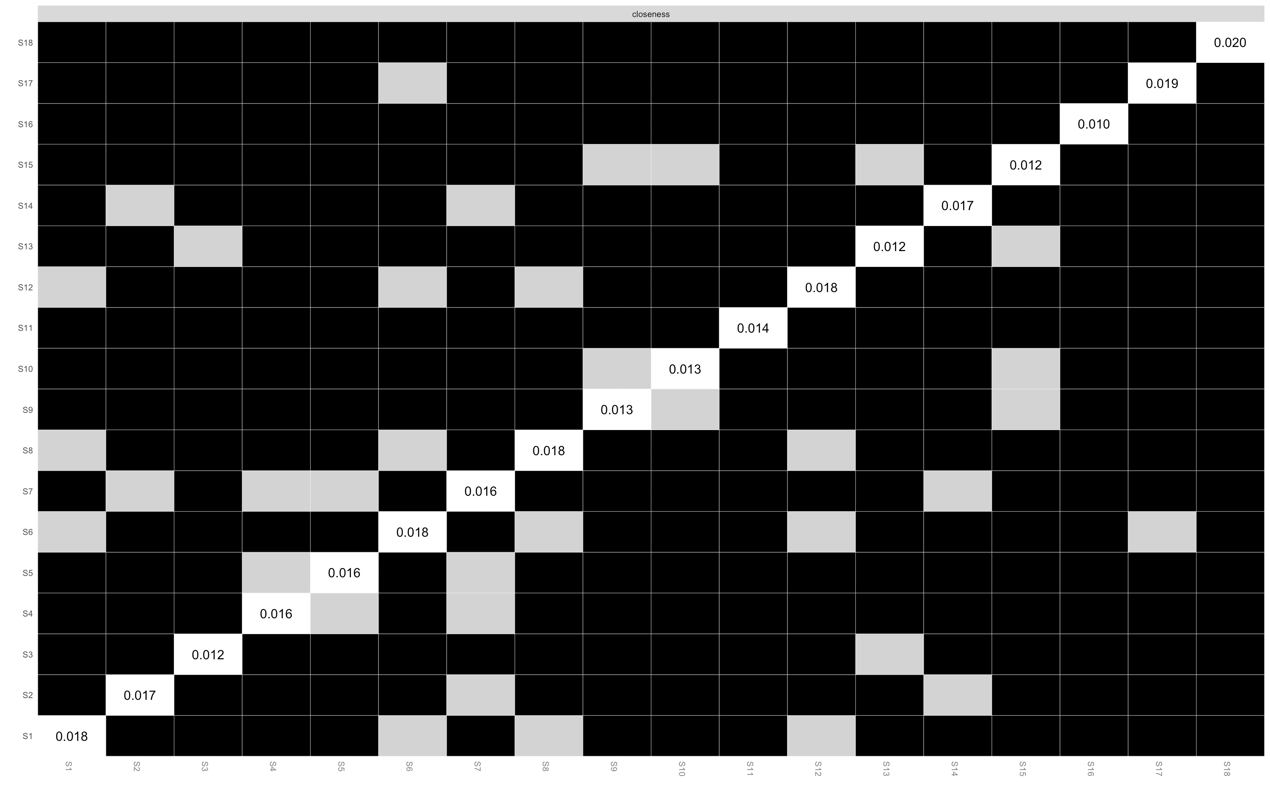


**Supplementary Figure 2: Estimation of node closeness differences by bootstrapped difference test**

**Note:** Nonparametric bootstrapped difference test for closeness. Gray boxes indicate no difference between nodes, whereas black boxes indicate significant difference (α = 0.05). Values reported in the diagonal represent the strength values of each node.

**
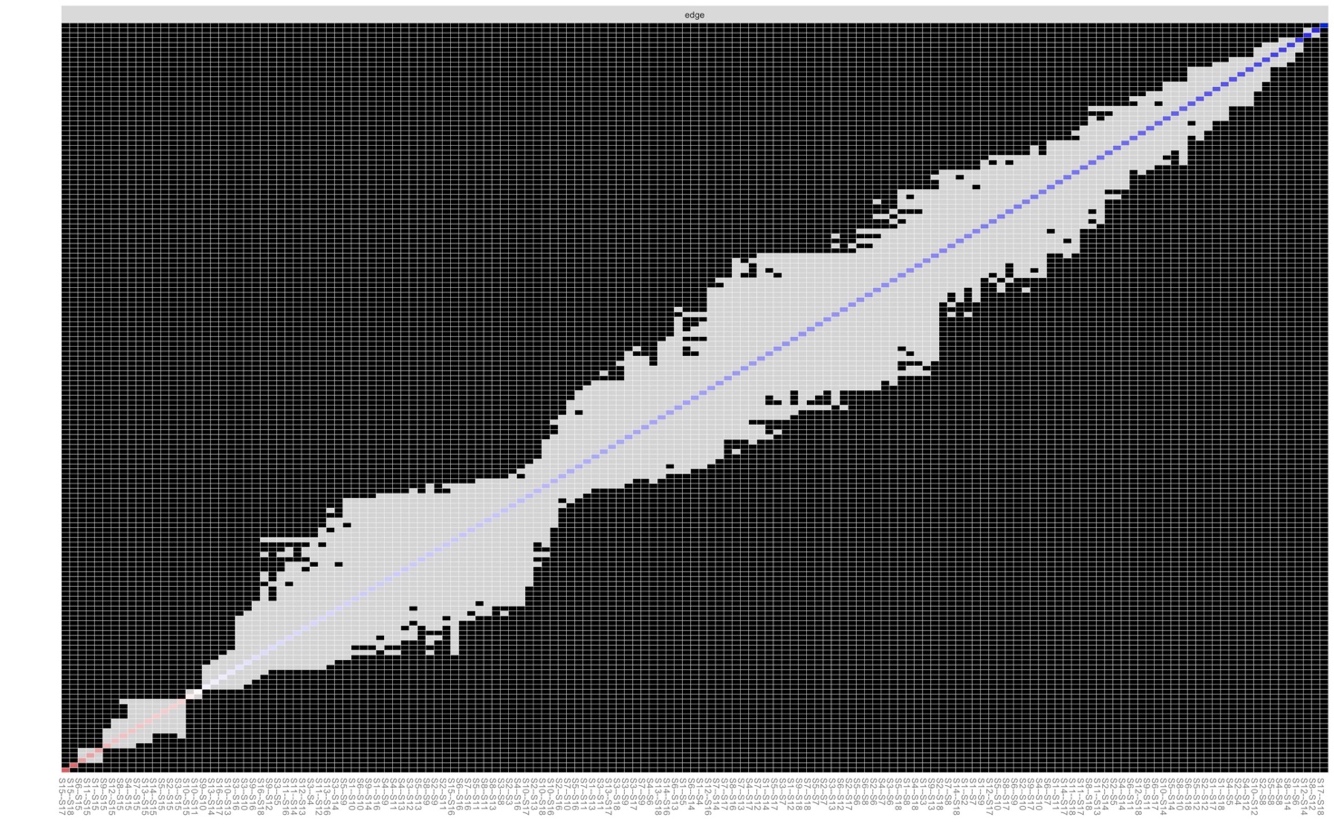
**

**Supplementary Figure3: Estimation of edge weight differences by bootstrapped difference test**

**Note:** Nonparametric bootstrapped difference test for edge weight. Gray boxes indicate no difference between nodes, whereas black boxes indicate significant difference (α = 0.05). Values reported in the diagonal represent the strength values of each edge.


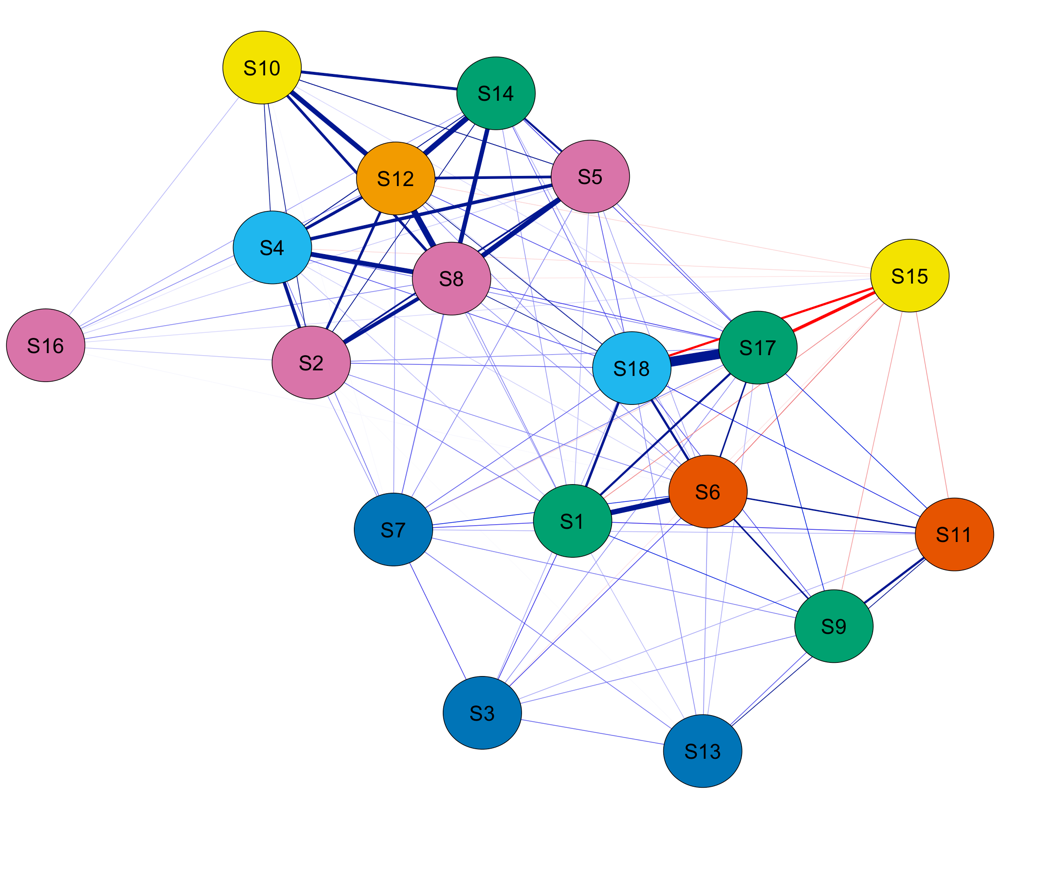


**Supplementary Figure 4: Network Analysis of Subjective Well-being of high School Boys**

**Note：**S1: How have you been feeling in general, S2: Have you been bothered by nervousness or you “nerves”, S3: Have you been in firm control of your behavior, thoughts, emotions or feeling, S4: Have you felt so sad, discouraged, hopeless, or had so many problems that you wondered if anything was worthwhile, S5: Have you been under or felt you were under any strain, stress, or pressure, S6: How happy, satisfied, or pleased have you been with your personal life, S7: Have you had any reason to wonder if you were losing your mind, or losing control over the way you act, talk, think, feel, or of your memory, S8: Have you been anxious, worried, or upset, S9: Have you been waking up fresh rested, S10: Have you been waking up fresh and rested, S11: Has your daily life been full of things that were interesting to you, S12: Have you felt down-hearted and blue, S13: Have you been feeling emotionally stable and sure of yourself, S14: Have you felt tired, worn out, used-up, or exhausted, S15: How concerned or worried about your health have you been How much do you care or worry about your health, S16: How relaxed or tense have you been, S17: How much energy, pep, vitality have you felt, S18: How depressed or cheerful have you been. The same below.

**
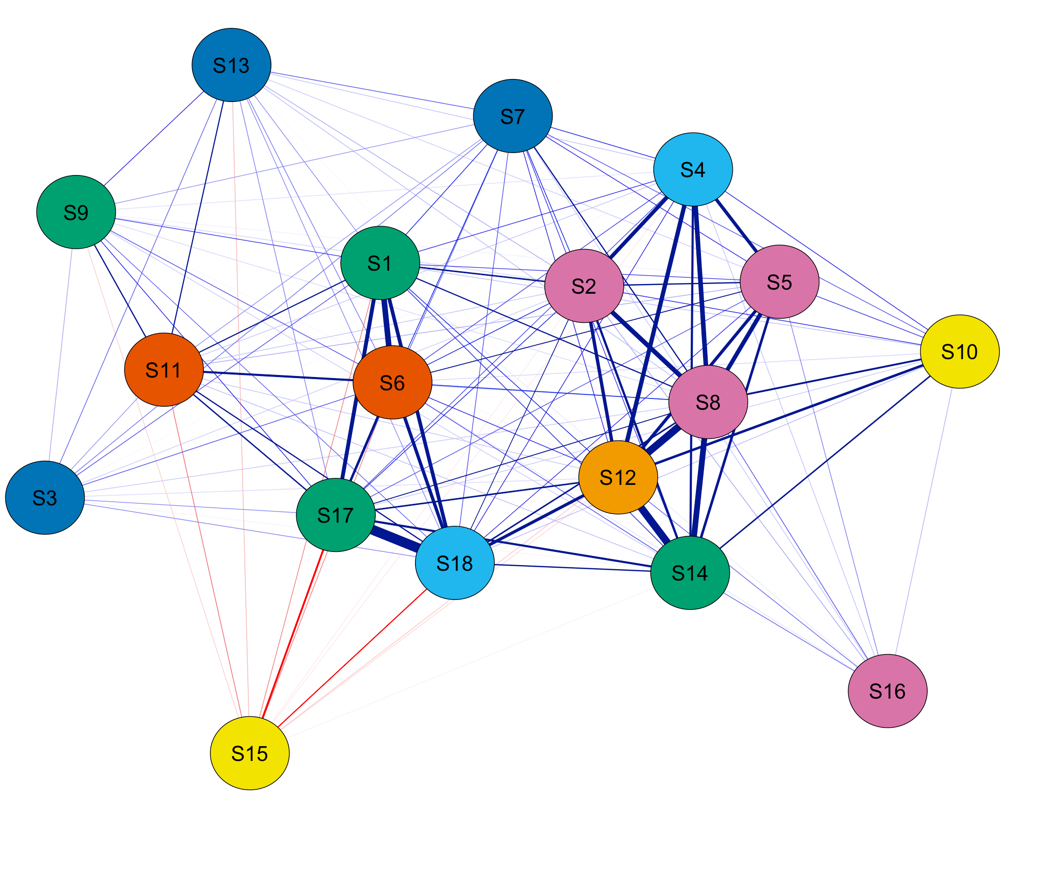
**

**Supplementary Figure 5: Network Analysis of Subjective Well-being of high School girls**

**
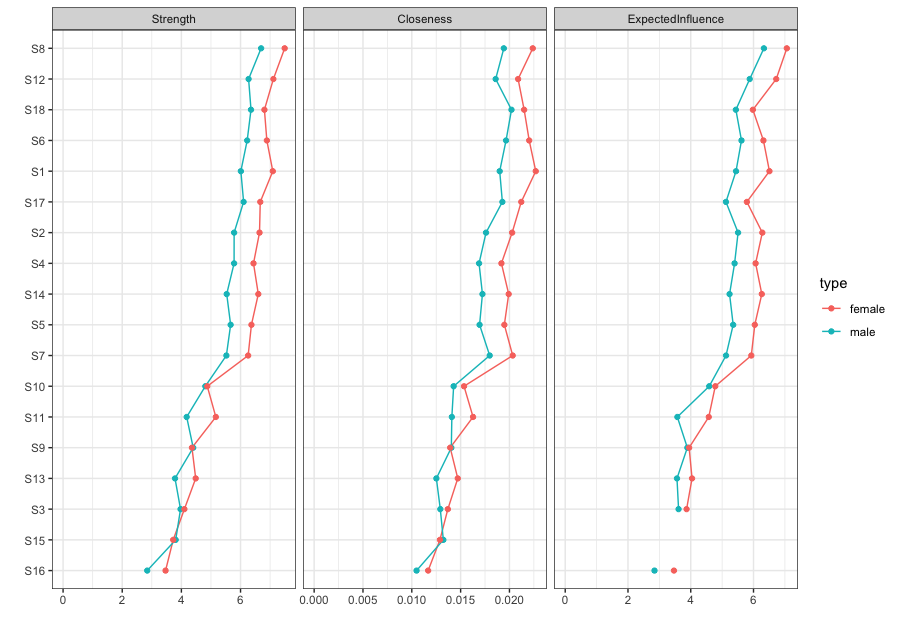
**

**Supplementary Figure 6: Node strength based on subgroups by gender**


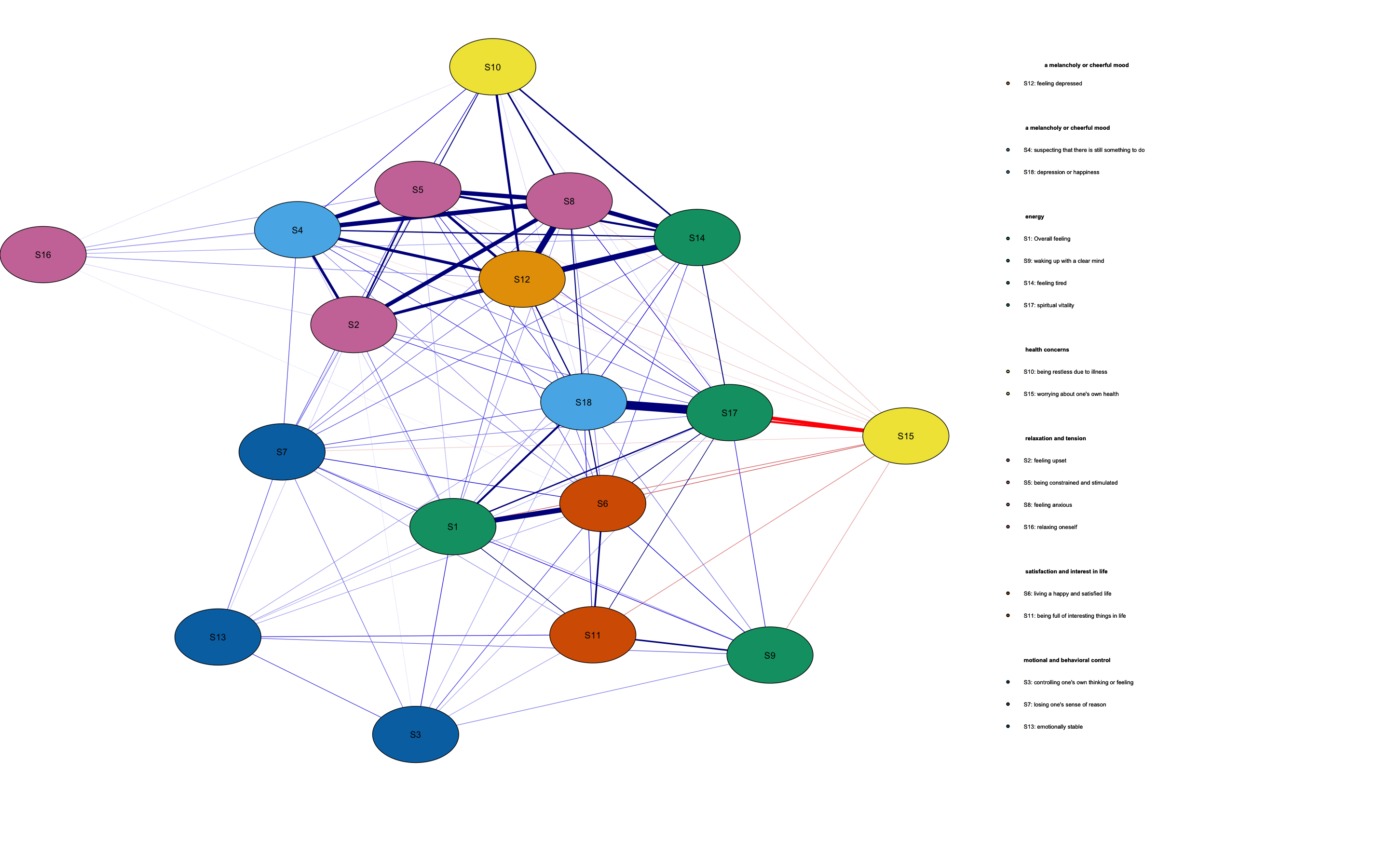


**Supplementary Figure 7：Network analysis of rural high school students' subjective well-being.**


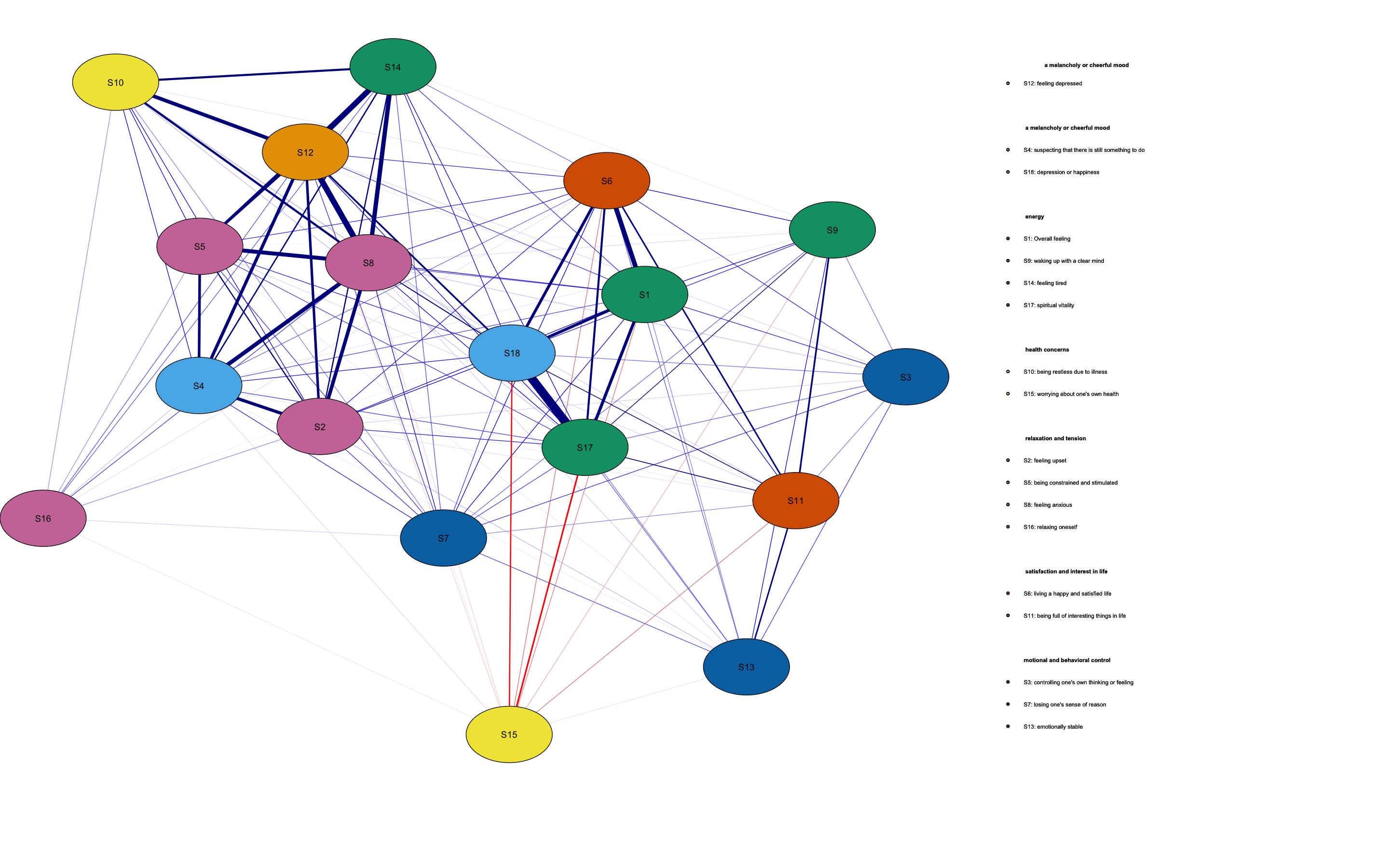


**Supplementary Figure 8：Network analysis of urban high school students' subjective well-being.**

**
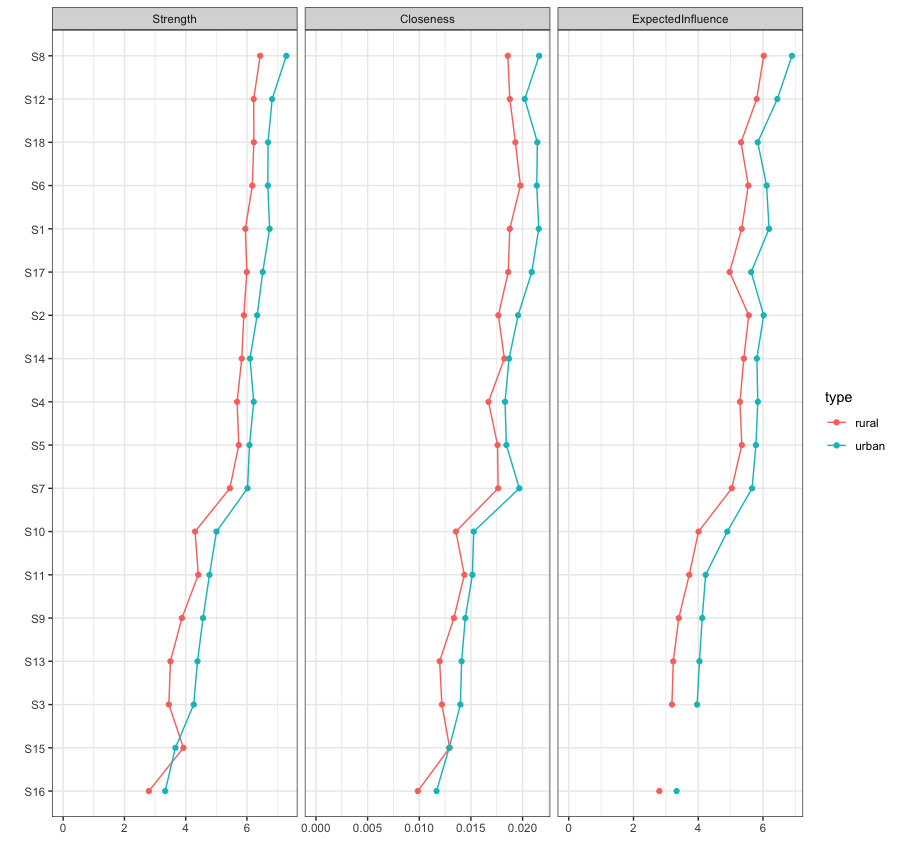
**

**Supplementary Figure 9：Node strength based on subgroups by residence.**
